# Survival and Population Dynamics of the Marabou Stork in an Isolated Population, Swaziland

**DOI:** 10.1371/journal.pone.0046434

**Published:** 2012-09-28

**Authors:** Ara Monadjem, Adam Kane, Andre Botha, Desire Dalton, Antoinette Kotze

**Affiliations:** 1 All Out Africa Research Unit, Department of Biological Sciences, University of Swaziland, Kwaluseni, Swaziland; 2 Department of Zoology, School of Natural Sciences, Trinity College Dublin, Dublin, Ireland; 3 Birds of Prey Programme, Endangered Wildlife Trust, Modderfontein, South Africa; 4 National Zoological Gardens of South Africa, Pretoria, South Africa; 5 Genetics Department, University of the Free State, Bloemfontein, South Africa; Australian Wildlife Conservancy, Australia

## Abstract

Investigating the ecology of long lived birds is particularly challenging owing to the time scales involved. Here an analysis is presented of a long term study of the survival and population dynamics of the marabou stork (*Leptoptilos crumeniferus*), a wide ranging scavenging bird from Sub-Saharan Africa. Using resightings data of tagged nestlings and free flying birds we show that the stork population can be divided into three general life stages with unique survival probabilities and fecundities. Fecundity of the storks is inversely related to rainfall during their breeding season. Corroborative evidence for a metapopulation structure is discussed highlighting the impact of the Swaziland birds on the ecology of the species in the broader region. The importance of tag loss or illegibility over time is highlighted. Clearly, any attempt at conserving a species will require a detailed understanding of its population structure, of the sort examined here.

## Introduction

Survival estimates are critical parameters for various ecological models, particularly population dynamics, which in turn inform conservation [1]. This is particularly important for long-lived vertebrates which may take several years to mature into breeding adults [2], [3]. However, survival estimates are often not available for long-lived birds such as storks; the only species for which robust estimates of both subadults and adults are available is the white stork *Ciconia ciconia* [4], [5], [6]. Survival in the marabou stork *Leptoptilos crumeniferus* has been crudely estimated by comparing the proportion of immature to adult birds [7], however robust estimates of survival must be based on following marked individuals through time [8].

**Figure 1:**
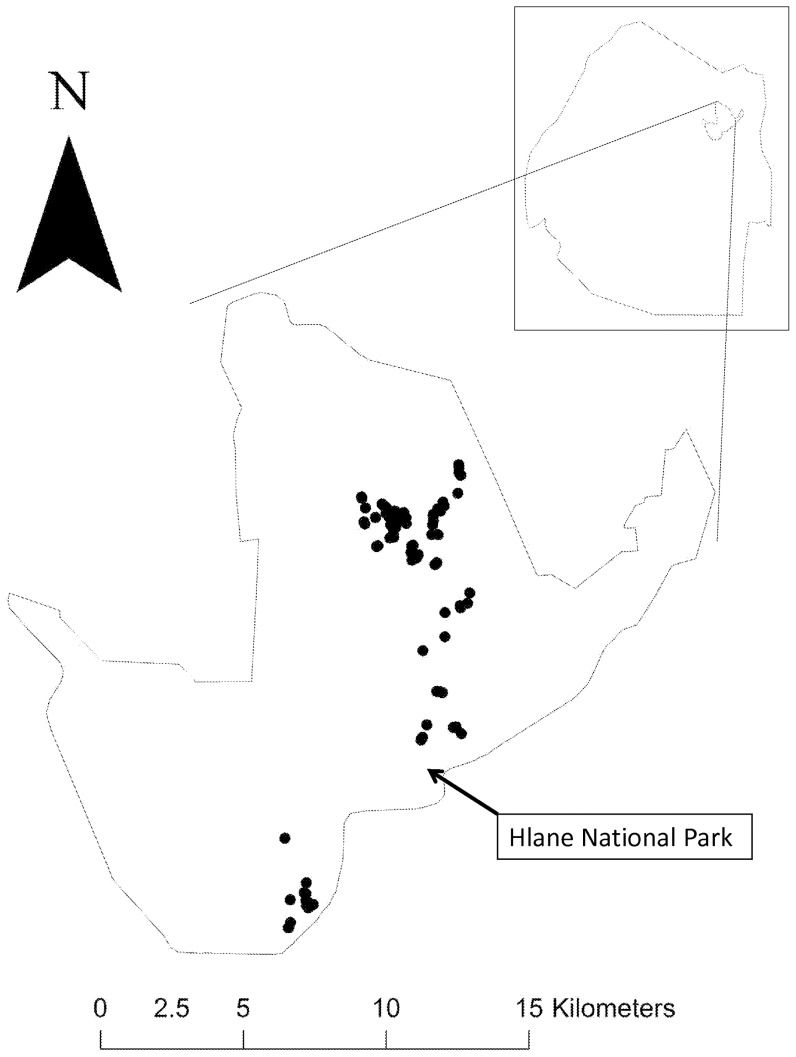
Map showing the distribution of marabou stork nests (black circles) at Hlane National Park. The inset shows the location of Hlane National Park within Swaziland.

**Figure 2:**
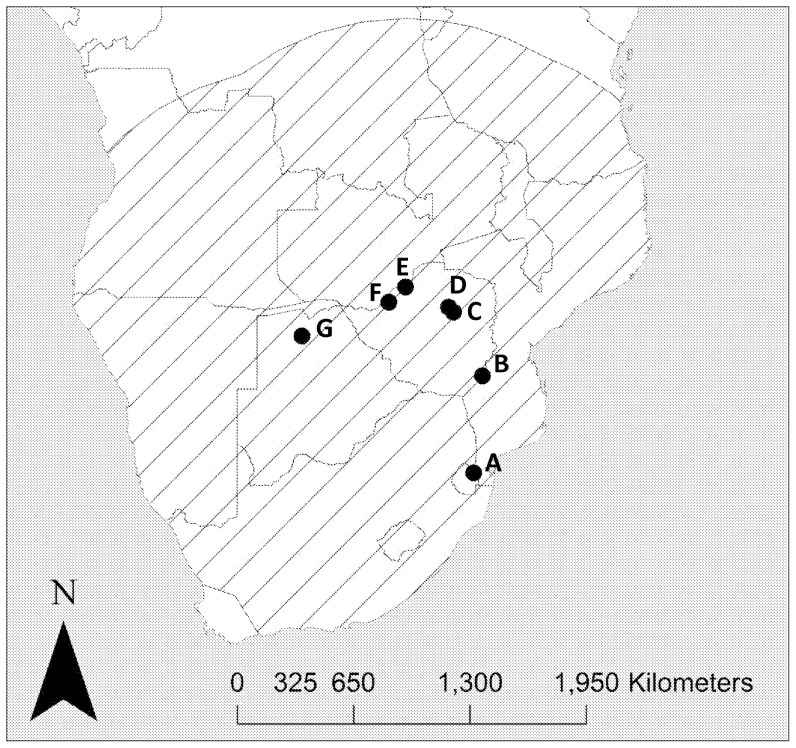
Map showing the distribution of marabou stork nesting colonies known to be active since 1990 in southern Africa (south of the Zambezi and Cunene rivers). The nesting colonies are represented by black circles and are as follows: (A) Hlane National Park, Swaziland [12]; (B) Gonarezhou National Park, Zimbabwe [14]; (C) Imbgwa Farm, Zimbabwe [14]; (D) Carswell Farm, Zimbabwe [14]; (E) Matusadona National Park, Zimbabwe [14]; (F) Binga, Zimbabwe [14]; (G) Okavango Delta, Botswana. The hatching represents all areas within 1500 km of these nesting colonies and therefore within dispersal distance of recently fledged chicks (see Discussion).

The marabou stork is a widespread scavenging bird occurring in savanna habitats throughout sub-Saharan Africa [9]. Despite its extensive distributional range, this species breeds at a rather limited number of localities [10]. For example, despite having been recorded across most of eastern, central and northern South Africa, it has not yet successfully bred in that country [11]. In fact, in all of southern Africa, this species breeds at just one site in Swaziland [12] (Figure 1), one site in Botswana [13] and less than a dozen scattered sites in Zimbabwe [14]. Hence this species has a metapopulation structure [15] with breeding birds concentrated in discrete colonies (Figure 2).This raises the possibility that the breeding populations act as sources for the South African sink. Dispersing birds can cover extensive distances of up to 1500 km thus allowing for dynamics of this sort to occur [10].

One way to infer a metapopulation structure for this species would be through a population dynamics model. Such models require a number of parameters for them to have any predictive power, notably probability of survival and fecundity rates [16]. Moreover, in most species these parameters will change as they age; a first year marabou stork, for instance, is not going to be involved in reproduction [17]. This is the shortcoming of exponential or geometric decay models of population growth which treat all life stages equally, resulting in a serious oversimplification [18]. An extensive knowledge of the breeding ecology and demographics of marabou storks is therefore necessary before such population dynamics modelling can be conducted.

The aim of this paper is to elucidate the population ecology of the marabou stork in southern Africa. It is hypothesized that survival is age dependent and that this species has a positive population growth rate. The objectives of this paper are to:

1. Relate fecundity of marabou storks breeding in Swaziland over a nine year period with climatic conditions,
2. Estimate survival rates based on resightings of tagged nestlings and free-flying marabou storks in southern Africa,
3. Use the breeding data and survival estimates of the breeding population in Swaziland to model the population dynamics of this species.

## Materials and Methods

### Study area

The collection of breeding data and the tagging of nestlings was conducted in the 16 000 ha Hlane National Park (31°53′S, 26°18′E), Swaziland. The vegetation is dominated by knobthorn *Acacia nigrescens* woodland interspersed with riverine forest. The climate is subtropical [see [12], [19] for more details on topography and climate]. The marabou stork breeding season covers the austral winter: 2–3 eggs are laid in May or June; eggs hatch after approximately 30 d incubation, usually in June or July, which is the coldest time of year; and the chicks fledge in October [20], [10], [19], [21].

Free-flying marabou storks were captured and tagged at the Moholoholo Wildlife Rehabilitation Centre (24°30′S; 30°54′E, 600 m above sea level), north-eastern South Africa. The site is situated in low-lying savanna, to the west of the Kruger National Park.

There is no Ethics Committee at the University of Swaziland to oversee the compliance of biological studies. However, the current study was conducted under a permit from the Swaziland National Trust Commission to Ara Monadjem. As outlined in our methods, the only contact we had with the study animals was tagging 210 birds based on a standard technique applicable throughout the World. Furthermore, we did not harm or compromise the health of any species during this study.

Patagial tagging was implemented as the preferred method of colour-marking for large raptors, vultures and storks by the Birds of Prey Programme of the Endangered Wildlife Trust in 2006. An extensive review and assessment process of a range of methods was conducted over a period of 18 months before the members and associates of the Programme agreed on and approved the tagging protocol for the use of this method at its Annual Conference [22]. The review included a clinical assessment of the condition of captive and recaptured free-flying tagged birds by independent veterinarians after tagging and no signs of infection or other potentially negative symptoms resulting from the tagging have to date been found [22]. Based on the results of the survival and movements of tagged birds, the method also does not seem to have a detrimental effect on the mobility and foraging ability of other large birds e.g. vultures [23].

### Data collection

Breeding marabou storks were monitored regularly between 2003 and 2011 at Hlane National Park. Originally nests were located on foot, but from 2008 we used a microlight to search for nests from the air once per year in July. Active nests (a nest on which eggs were found or adult activity was observed) were visited twice weekly until the chicks hatched or the nest failed. Fledging date was taken as the date of the last visit on which the chick was still on the nest. The mass and wing length was measured during each visit.

Free-flying birds were captured in a specially designed walk-in trap for vultures [24], [25] that was erected alongside the vulture restaurant at Moholoholo Wildlife Rehabilitation Centre. Captured birds were aged and had their mass and wing length measured. Ageing was based on plumage characteristics [26] and birds were assigned to one of three age classes: juvenile (1^st^ year bird), subadult (2–4 years old) and five years old or older. Each bird was fitted with a metal ring issued by AFRING (Animal Demography Unit, University of Cape Town) and a patagial tag. Patagial tags were fitted according to the standard protocol adopted for this practice in southern Africa [22]. It involved the use of a double set of standard cattle tags engraved with a unique number which was fitted to the patagial area on each wing of each bird using a tag applicator. This method was extensively assessed prior to this study and found not to be detrimental to the birds' health or inhibiting their ability to forage [22]. All tagged marabou storks were released unharmed and within 120 min of capture.

A dedicated resightings programme was established using radio and television broadcasts, newspaper and magazine articles, and posters in Kruger National Park rest camps. A significant proportion of resightings was submitted by the staff at Moholoholo who kept a daily watch at the vulture restaurant, at which the marabou storks were frequently observed. Resightings were also reported inter alia by managers of other vulture restaurants, game ranchers, farmers and tourists.

### Sexing

Only nestlings were sexed. DNA extraction was conducted using the QiagenDNeasy® Blood and Tissue Kit. The extraction protocol as outlined in the manufacturer protocol was followed. CHD1 gene amplification was conducted using the 2550F/2718R [27] primer set at the National Zoological Gardens of South Africa. Amplification was carried out using 25 µl reaction volume and polymerase chain reaction was conducted with PromegaGoTaq® Flexi DNA polymerase (Promega Corporation) which has a 1× buffer containing 10 milli molar (mM) Tris®­HCl (pH 9.0), 50 mM potassium chloride (KCl) and 0.1% Triton® X­100. The final reaction conditions were as follows: 1 X PCR buffer, 1.5 mM MgCl2, 200 micro molar (µM) of each 2′­deoxynucleotide triphosphate (dNTP), 5 picomol (pmol) of each of the forward and reverse primer, 0.25 unit (U) Taq DNA polymerase and 10–20 nano gram (ng) genomic DNA template. A no template control as well as positive controls for a male and female bird of known sex was included. The conditions for PCR amplification were as follows: 2 min at 95°C initial denaturation, 30 cycles for 30 s at 95°C, 30 s at 50°C and 2 min at 72°C, followed by extension at 72°C for 10 min. The PCR reaction was carried out in the BOECO TC-PRO Thermal Cycler. PCR products mixed with tracking dye were separated by electrophoresis in a 2% agarose gel for 45 min at 100 V in 1× Tris-borate- EDTA buffer.

### Data analysis

Fecundity is a measure of reproductive success and was defined as the number of fledglings successfully raised per pair per annum [28]. A Pearson's correlation was used to test whether fecundity was related to rainfall in the preceding summer (October to March, inclusive) and rainfall during the winter breeding season (May to September), as these variables had previously been shown to be important for marabou stork breeding [19].

The program MARK was used to estimate survival and recapture of marabou storks using the standard Cormack-Jolly-Seber model [29], [30]. A variety of models that included time dependence, sex and age were developed. Models were ranked using Akaike's Information Criterion corrected for small sample size (AIC_c_) [31]. The model with the lowest AIC_c_ was deemed the best model; where ΔAIC_c_ for any two (or more) models was <2.0, they were both deemed to be equally good.

Survival was estimated separately for the birds tagged as nestlings from those tagged as free-flying. A subset (n = 100, tagged between 2008 and 2011) of those tagged as nestlings were sexed, and were used to test for the role of sex in survival of marabou storks. Models which included sex performed poorly compared with those that did not (ΔAIC_c_>2.9) and hence sex was removed as a factor. Subsequently, the data for the sexes of the nestlings were pooled and only age and time dependence were included.

To test for violations of the assumptions of homogeneity of survival and recaptures, GOF (goodness of fit) tests were conducted in the program Release [32]. Test 2 tests the assumption of equal catchability (in our case, resightings) of marked individuals. Test 3 tests the assumption that all individuals have equal probability of survival independent of when they were marked.

### Model Building

Leslie Matrices are used to chart the development of a population over time by separating the given group into distinct age classes, with matrix elements representing probability of survival and fecundity of these classes [33]. A variation of a Leslie Matrix was developed to investigate the population dynamics of a marabou stork population. The model used information from females only as they are responsible for reproduction in the population [34] and assumes that the above parameters are constant for each age class. The number of females in each class is given by a column vector **N**_t_ such that the number determined at time t+1 is given by **N**_t+1_ = **LN**_t_, where L represents the Leslie Matrix. This process is repeated for each time step. In this case t represents one year. This gives the following general equation:
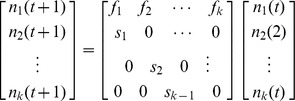
where“f” is fecundity and “s” is the probability of survival from one age class to the next. The dominant eigenvalue of the matrix is the λ value and represents the growth rate of the population. If λ>1, the population is growing, 0<λ<1, it is decreasing and λ = 1, the population is stable. The right eigenvector of a given matrix represents the stable age distribution of the group i.e. the proportion of birds in each stage. This is a measure of the contribution the life stages have to overall population growth [35]. So although juveniles do not reproduce they still make a contribution here.

A variation of the Leslie Matrix known as the Lefkovich Matrix was used in order to separate the storks into distinct life stages [16]: juveniles, subadults and adults,
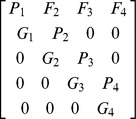



Gx (on the subdiagonal) is the probability of surviving for a year and moving into the next stage; Px (on the diagonal) is the probability of surviving for a year and remaining in the same stage; Fx represents fecundity. The model did not take into account density dependent effects. The matrix was created in MS Excel using a variation of a template developed by Spangenberg and Jungck [33] (see results section for the values used to parameterize the model). Seeing as subadult and adult birds can remain in their respective stages for more than a single year this was corrected for accordingly with the following equations,
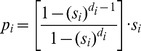


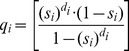



The probability of remaining in an age class at next year is given by p_i_ and q_i_ is the probability of moving up an age class at next year. S_i_ is the survivor rate for i^th^ year and d_i_ is the length of time spent in this i^th^ stage (taken from [16]).

**Figure 3:**
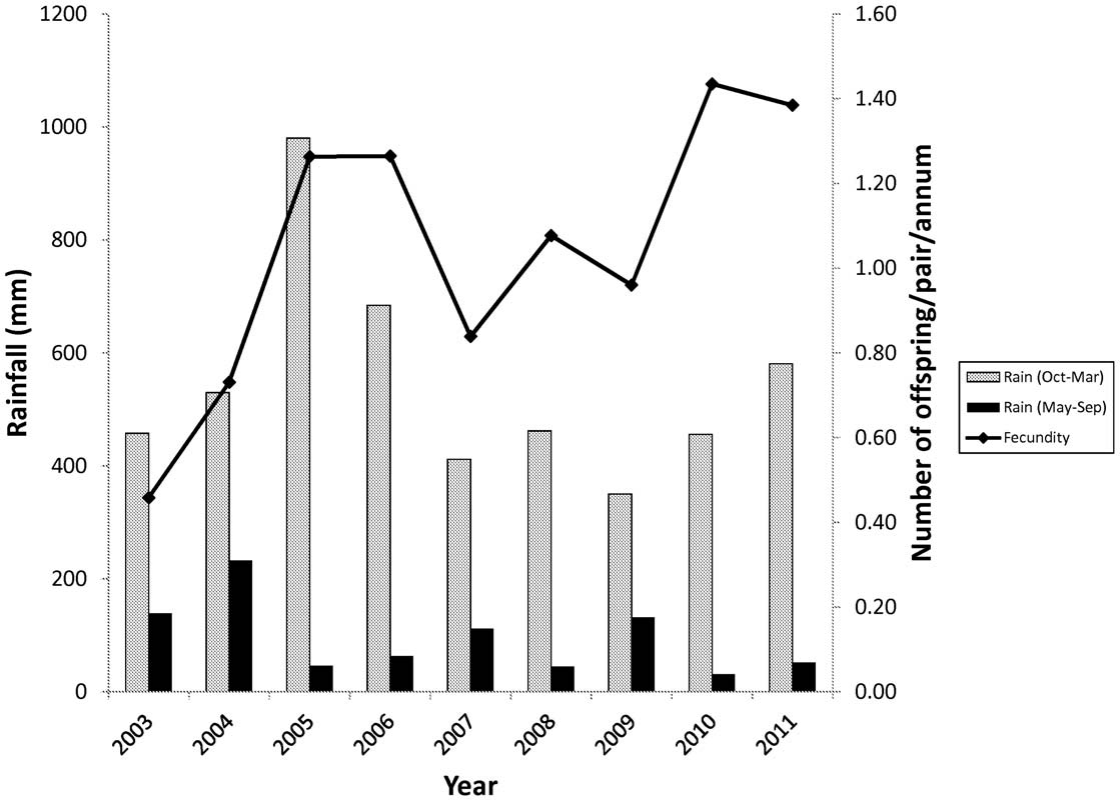
The relationship between fecundity of marabou storks and rainfall between 2003 and 2011 in Swaziland.

The below matrix was developed using the values from our results which corrected the original parameters for stage duration and fecundity,
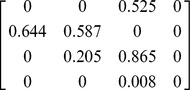



This matrix has the three defined age classes i.e. the columns (there is a small non-zero probability that the birds will live past the third stage and fall into a fourth category but any individuals here have zero probability of surviving to the next time step). Subadult birds spend years two to four in their stage and adults spend from age five to 25 in their stage. This assumes a life expectancy of 25 years for wild marabou storks [36], [11]. The fecundity value shown here was taken as an average from 2003–2011 (see Figure 3). The model was run with a varying starting population of three to 10 adult females; the lower number represents a minimum estimate of the nesting population in Swaziland during the early 1960s [37]. However, seeing that nesting sites can easily be overlooked by observers on the ground we also ran the model with a starting population of up to 10 females.

## Results

Between 19 and 31 pairs of marabou storks bred annually at Hlane National Park that fledged between 11 and 43 chicks. The fecundity of marabou storks breeding at Hlane National Park differed greatly between years (Figure 3), with a mean of 1.05 fledged offspring per pair per annum. There was no correlation between fecundity and rainfall in the previous summer (r = 0.401, df = 7, P = 0.284), however, there was a significant inverse correlation with rainfall during the breeding season (r = −0.785, df = 9, P = 0.012).

A total of 193 nestlings and 17 free-flying marabou storks have been tagged since 2005 and which have been resighted 811 and 834 times, respectively. On fledging, male marabou storks had significantly larger mean (± SE) wing lengths (663.1±4.63 mm vs 596.1±5.85 mm; t = 8.98, P<0.0001, DF = 98) and heavier mean (± SE) masses (7437±110 g vs 6155±114 g; t = 8.09, P<0.0001, DF = 98) than females. Free-flying birds had mean (± SE) wing length of 700.7±7.88 mm (n = 17), and mean (± SE) mass of 5038±291 mm (n = 13).

In the nestling analysis covering the seven years between 2005 and 2011, survival of nestlings post-fledging was age-dependent. The best model for survival had three age classes, 1^st^ year birds, 2^nd^–4^th^ year birds and ≥5^th^year birds (Table 1). The next model had ΔAIC_c_<2 compared with the best model, and had survival separated into five age classes. For both of these models recapture rates were age and time independent (Table 1). The next three models all had survival being age dependent. The first model to have survival independent of age had a ΔAIC_c_>5 (Table 1). All the test 2 and test 3 results were statistically not significant (chi-square test, P>0.05), showing that the assumptions tested had not been violated.

**Table 1:** The candidate models used to estimate survival in nestling marabou storks tagged in Swaziland between 2005 and 2011, and resighted across southern Africa.

Model	AICc	Delta AICc	AICc Weights	N
phi(age1, 2–4, ≥5) p(.)	489.751	0	0.13917	4
phi(age1, 2, 3, 4, ≥5) p(.)	490.1439	0.3928	0.11435	5
phi(age1, 2, 3, 4, ≥5) p(age1, ≥2)	492.1082	2.3572	0.04282	6
phi(age1, 2, 3, 4, ≥5) p(t)	494.1198	4.3688	0.01566	10
phi(age1, ≥2) p(.)	494.3246	4.5736	0.01414	3
phi(t) p(.)	495.4601	5.7091	0.00801	7
phi(age1, 2, ≥3) p(.)	496.0157	6.2647	0.00607	4
phi(t) p(t)	496.0794	6.3284	0.00588	10
phi(age1, ≥2) p(age1, ≥2)	496.2424	6.4914	0.00542	4
phi(age1, 2, 3, 4, ≥5) p(.)	497.811	8.0600	0.00247	5
phi(age1, ≥2+t) p(.)	500.4836	10.7326	0.00065	11
phi(age1–2, ≥3+t) p(.)	502.8863	13.1353	0.0002	11
phi(age1, 2, ≥3+t) p(.)	505.8063	16.0553	0.00005	15

Estimates of survival (phi) and recapture (p) were modelled with time (t), and/or age class of the birds (age). Age1–5 refers to age classes of 1^st^ year birds through to 5^th^ year birds. The number of parameters is indicated by “n”. The models are arranged from best (top of table) to worst (bottom).

In the analysis of the free-flying birds, the best model had survival separated into two age classes (Table 2). In this model, the two age classes were 1^st^ year birds, and subadult/adult birds (i.e. no difference in survival of subadults and adults). The next three best models all had ΔAIC_c_<2, suggesting that they were not statistically distinguishable from the best model. In all three of these models survival was separated into three age classes. In the top three models, recapture rates were age dependent, whereas in the fourth best model recaptures were independent of both age and time (Table 2). Again, the test 2 and test 3 results were statistically not significant (chi-square test, P>0.05), showing that the assumptions tested had not been violated.

**Table 2:** The candidate models used to estimate survival in free-flying marabou storks tagged in South Africa between 2007 and 2011, and resighted across southern Africa.

Model	AICc	Delta AICc	AICc Weights	n
phi(age1, ≥2) p(age1, ≥2)	52.0191	0	0.29804	3
phi(age1, 2, ≥3) p(age1, 2, ≥3)	52.554	0.5349	0.22810	4
phi(age1, 2, ≥3) p(age1, ≥2)	52.5604	0.5413	0.22737	4
phi(age1, 2, ≥3) p(.)	53.2647	1.2456	0.15988	4
phi(age1, 2, ≥3) p(t)	59.7627	7.7436	0.00621	6
phi(age1, 2, ≥3+t) p(age1, 2, ≥3+t)	66.2284	14.2093	0.00024	8
phi(age1, 2, ≥3 *t) p(age1, 2, ≥3 *t)	79.696	27.6769	0	11

Estimates of survival (phi) and recapture (p) were modeled with time (t), and/or age class of the birds (age). Age1 refers to age classes of 1^st^ year birds, age2 to subadults (2^nd^ to 4^th^ year birds) and age3 to adults (≥5^th^ year birds). The inclusion or exclusion of interactions in the models are symbolized by (*) or (+), respectively. The number of parameters is indicated by “n”. The models are arranged from best (top of table) to worst (bottom).

The survival rates of marabou storks varied considerably between age classes and the datasets analysed (Table 3). Typically, first year survival was lower than that of older birds. Based on the analysis of birds tagged as nestlings, survival to the end of the first year was 0.6440. However, based on the analysis of free-flying birds, the survival of 1^st^ year birds was only 0.2500. Survival of older age classes (subadults and adults) was generally high ranging from 0.7917 to 0.8727 (Table 4). The only exception was ≥5^th^ year birds tagged as nestlings where survival dropped to 0.2193, suggesting loss or fading of tags.

**Table 3:** Survival and recapture (resightings) rates of marabou storks tagged as nestlings and free-flying adults in South Africa between 2005 and 2011.

Analysis	Estimated parameter	rate	SD
Nestlings	Survival of 1^st^ year birds	0.6440	0.0765
Nestlings	Survival of 2^nd^–4^th^ year birds	0.7917	0.0597
Nestlings	Survival of ≥5^th^ year birds	0.2193	0.1459
Nestlings	Recapture rate	0.4226	0.0523
Free-flying	Survival of 1^st^ year birds	0.2500	0.2165
Free-flying	Survival of ≥ subadult birds	0.8727	0.2483
Free-flying	Recapture of 1^st^ year birds	0.9999	0.0004
Free-flying	Recapture of ≥ subadult birds	0.2545	0.1559

The analysis refers to the specific dataset used for the estimates: nestlings = all nestlings tagged and resighted between 2005 and 2011; free-flying = all free-flying birds tagged and resighted between 2007 and 2011. See Methods for further details.

**Table 4:** Parameters used in matrix.

Stage	Fx	Px	Gx
Juveniles	0	0	0.644
Subadults	0	0.587	0.205
Adults	0.525	0.865	0.008

Fx = Fecundity; Px = Probability of remaining in age class at next year; Gx = Probability of moving up an age class at next year.

**Figure 4:**
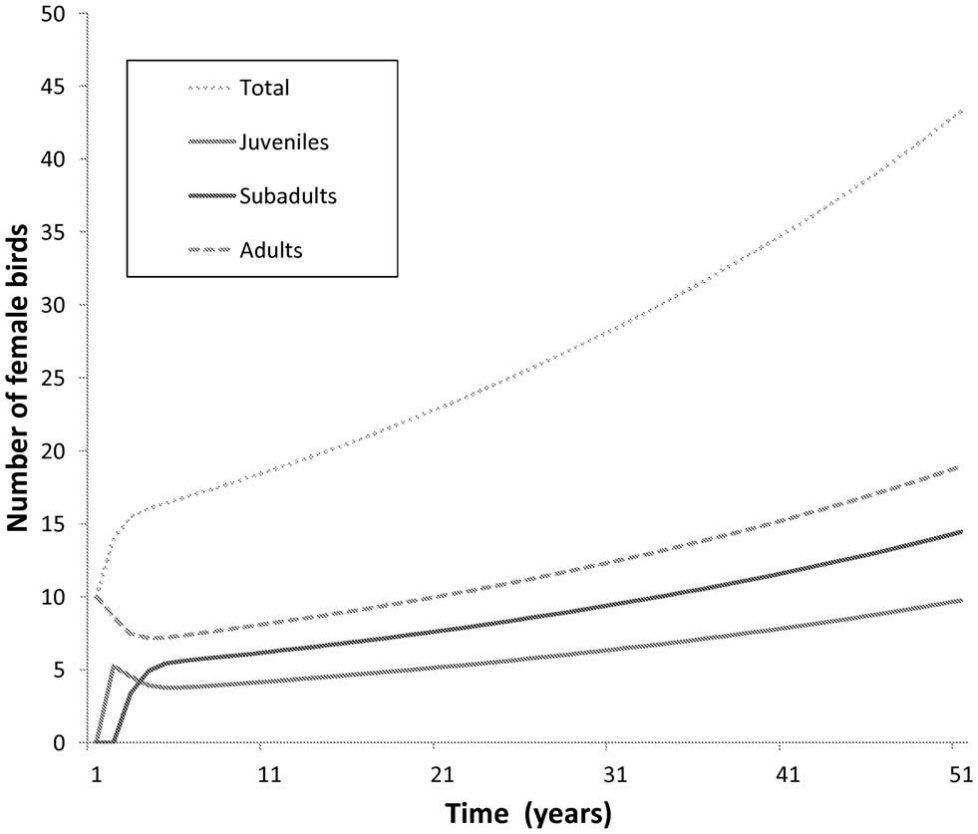
Graph showing the growth of the total population and each component stage over a 50 year period commencing in 1962. The initial population was set to 10 females in this case. λ = 1.0212.

The resulting λ value was 1.0212 indicating a population increase over time (see Figure 4). The population in this model developed from exclusively adults to an age structure which stabilizes at 43.8% adults, 33.4% subadults and 22.5% juveniles (Table 5). The reproductive value increases with age in marabou storks with the adult stage being the highest (see Table 5).

**Table 5:** Values for the stable age distribution and the reproductive value of the life stages as given by the right and left eigenvectors of the matrix respectively.

Stage	Stable Age Distribution	Reproductive Value
Juveniles	0.2251	1.00
Subadults	0.3337	1.59
Adults	0.4378	3.36

The final population in the model is clearly dependent on the initial number of founding individuals and the fecundity of the storks. Starting with three females a total population of 26 birds is produced over the 50 year time frame; 10 females resulted in 86 birds (Figure 4). The total number of adult females was six and 19 respectively (Figure 4).

## Discussion

This study presents the first estimates of survival for the marabou stork based on resightings of tagged individuals that also accounts for recapture (resightings) probability. A previous study estimated the survival of marabou storks based on the proportion of immature birds to adult birds, yielding survival rates of 28% for first year birds, 72% for subadults and 92% for adults [7], [38]. Our estimates are lower than these for adults but higher for juvenile and subadult birds. Our estimate for sub-adult survival mentioned above also matches those for the wood stork, *Mycteria americana* [39]. From the tagging of nestlings, our estimate of 1^st^ year mean survival is 64%. This is higher than survival rates for 1^st^ year white storks estimated at 47% [40], 48% [4] and 33–42% [41], and for 1^st^ year wood storks at 44% [42]. This is not surprising since the marabou stork is larger than these two species and survival rates are directly related to size [38]. However, the estimate of 1^st^ year survival based on the tagging of free-flying birds was exceptionally low (25%), lower than that reported for the white or wood storks. Undoubtedly, this estimate suffers from small sample sizes as only four free-flying 1^st^ year birds were captured and tagged compared with 193 nestlings.

Adult survival was higher, but the specific value depended on the dataset that was used. Based on the free-flying birds the survival rate was 87% and apparently did not differ between subadults and adults. Subadult (2^nd^–4^th^ year birds) mean survival rates based on resightings of tagged nestlings was 79%, with adult (≥5^th^ year birds) survival rate dropping to 22%. This clearly is not an accurate reflection of adult survival, where captive marabou storks may live to 31 years [11], but may be related to tag loss (see below). The annual survival of adult white storks in Europe exhibited considerable inter-annual variation over a 19-year period ranging from a low of about 70% to almost 100% [41]. By contrast, mean annual survival rates in declining populations of white storks in Europe ranged between 58% and 75% [4].

Survival rates of the marabou stork were not time-dependent. In contrast, the variation in survival rates of the white stork has been linked to rainfall in the Sahel where this species migrates to in the northern winter [40]. The marabou stork is not a migrant [11] which may explain the lack of time-dependence in this species. The lack of time-dependent survival, however, is still surprising in the marabou stork, especially since reproductive success of this species is strongly related to rainfall [19]. The current study corroborated the findings of a previous study [19] showing fecundity to be negatively related to rainfall during the breeding season. The reasons for this have previously been argued to be related to food availability and foraging efficiency of the parents [19] rather than due to the inability of the chicks to thermoregulate in cold and cloudy conditions [43]. Marabou storks are scavengers feeding on a wide range of food resources including carrion from large mammal carcasses, aquatic vertebrates and human waste [7], [17], [44]. The exact nature of the relationship between rainfall, food and marabou stork fecundity remains unclear, but we suggest the following. Since rainfall is necessarily linked with cloud cover, increased rainfall during the breeding season represents increased cloud cover during this period of food stress when parents need to fend for themselves as well as growing chicks. However, marabou storks require thermals for foraging [17], which are only available on sunny days, and hence increased cloud cover may result in less soaring time and less efficient foraging.

There are a number of hypotheses that have been put forward to explain the relationship between rainfall and breeding success. One suggestion is that high rainfall impedes the formation of thermals on which the Marabous soar while they forage [19]. Another is that rainfall impacts on breeding success indirectly by influencing food availability for the storks. During the breeding season freshwater fishes make up a significant portion of the storks' diet [44]. Indeed the nestlings were observed to regurgitate fish on which they had recently been fed [19]. Low rainfall would grant the birds easier access to fish stocks as they wade through rivers and streams with lower water levels [19].

There was no apparent difference in the survival of male and female marabou storks. A similar result has been reported for the white stork [4]. However, female wood stork fledglings had survival rates of up to five times higher than in the larger males [41]. In line with a previous study [21], male marabou storks had larger masses and wing lengths than female storks. Larger size may be correlated with larger mortality, especially under difficult environmental conditions [45]. This, however, was not the case in this study.

Tag loss or fading is a serious violation of the assumptions of capture-mark-recapture studies [46], [47]. In our study, tag loss seems to have been a factor in the older age classes (≥5^th^ year birds). A similar resightings study of African white-backed vultures fitted with the same tags showed that they were fading and become illegible from about 5–6 years of age and older [23]. In this study, we report a sudden and significant drop in survival rate from the age of 5 years and above, suggesting that the same fate befell these tags. Hence our survival estimates are only applicable up to the 5^th^ year of a bird carrying the tags. We suggest that future studies investigate the possibility of using more durable tags for tagging of marabou storks.

Pastor [18] proposes that source and sink dynamics could be explored using stage-structured models like the one used here. If two subpopulations are linked one would expect opposite lambda values because one population is supplying the other. Given the proposal here that Swaziland is the source, an investigation of the Kruger population could confirm this hypothesis. The fact that marabou storks have yet to breed successfully within the Kruger area [48] supports this statement. A positive correlation between breeding success and colony size for marabou storks has been reported [17]. Birds that bred successfully were typically in close proximity to other breeding pairs, often in the same tree [17]. So there may be Allee effects [49] at play in marabou nesting sites. This may explain the number of reported failed nests in Kruger National Park where breeding attempts involved single breeding pairs.

The lambda value from the matrix produced a population that either underestimated the modern day population completely or hit the lower range of the known breeding population encountered today of 19 pairs. This is with three and 10 breeding females respectively.

In general, this may be indicative of a metapopulation structure for marabou storks whereby more birds must be recruited to the local population in order to produce the numbers seen today [50]. The only data required to determine this are a local census and the λ [50]. So this is a potential way of predicting metapopulations with relatively little information. As noted above, one proviso is the sensitivity the model shows to the fecundity value, which varies year on year.

Specifically the lambda values reported here show that the Swaziland population of marabou storks is increasing. The metapopulation structure of marabou storks in southern Africa could explain the presence of a large population (300–400 individuals) of non-breeding birds in the Kruger National Park, South Africa [51]. The rate of increase seen in Swaziland is not enough to account for an assemblage of this size over a reasonable time period but if the breeding sites in Botswana and Zimbabwe are considered alongside (assuming similar population dynamics) the South African numbers could be explained. Juvenile marabou storks are capable of dispersing up to 1500 km after fledging, easily allowing them to cover the relatively short distance (<100 km) between the study site in Swaziland and Kruger National Park in South Africa [10] (see Figure 2).

The high reproductive value of adult marabou storks is worth highlighting. This value represents the contribution that birds at any stage make to population growth. Our analysis showed that adults contributed twice as much as subadults and over three times as much as juveniles to the growth of the population. The population growth of wood storks was similarly shown to be highly sensitive to adult survivorship [39]. Clearly, this is an important point to recognize for conservation efforts targeting long-lived species [52]. The inverse relationship between survival and reproductive value means that the emphasis should be on ensuring the survival of adults.
